# The Impact of the Different Stages of COVID-19, Time of the Week and Exercise Frequency on Mental Distress in Men and Women

**DOI:** 10.3390/nu14132572

**Published:** 2022-06-21

**Authors:** Lina Begdache, Anseh Danesharasteh, Zeynep Ertem

**Affiliations:** 1 Health and Wellness Studies, Binghamton University, Binghamton, NY 13902, USA; 2 Systems Science and Industrial Engineering, Binghamton, NY 13902, USA; adanesh1@binghamton.edu (A.D.); zeynep@binghamton.edu (Z.E.)

**Keywords:** exercise frequency, COVID-19, mental distress, gender, customization

## Abstract

The COVID-19 pandemic produced life disturbances and loss of routine which affected diet and sleep quality as well as physical exercise frequency. Interestingly, mental distress was higher even in those who exercised. The purpose of this study was to assess exercise frequency in relation to different levels of mental distress severity in men and women while accounting for working days and weekends. A de-identified secondary data set was analyzed. Regression analyses produced models of the different stages of COVID-19 in relation to physical exercise frequency and mental distress levels. Margin analysis generated predictive models that could be used prophylactically to customize physical exercise frequencies in men and women to reduce their risk of mental distress during future pandemics. Mental distress during the lockdown and after ease of restrictions was associated with different physical exercise frequencies, with a noticeable difference between men and women. During a pandemic, sedentary men are more likely to be mentally distressed during working days. Nevertheless, moderately active, but not very active women, may be less distressed during pandemic weekends. These findings may provide a framework to optimize mental health during different stages of a pandemic by customizing physical exercise frequencies based on gender and time of the week.

## 1. Introduction

Regular physical exercise is typically associated with enhanced physical health [1] and is considered an effective non-pharmacological modulator of mental health [2,3]. The benefits of physical exercise are mostly attributed to the improved blood flow to the brain, which promotes oxygen and nutrient delivery to neurons. In addition, physical exercise modulates brain chemistry by boosting serotonin, dopamine, and endorphin levels, which regulate mood. However, this boost in neurotransmitters appears to be only related to voluntary exercise (VE), or motivated exercise. VE also stimulates the release of brain-derived neurotrophic factor (BDNF), which supports neurogenesis and neuroplasticity that contribute to mental wellbeing as well [4]. Interestingly, involuntary exercise seems to reduce neurotrophic factors more so in females than males [5]. However, physical exercise and a good quality diet typically go together. In addition, physical exercise mediates the effect of food on mental health by enhancing the positive effect of nutrients and reducing the negative effect of low-quality food [6]. Several nutrients were described to support brain functions. Fruits, vegetables, nuts, fish, good quality proteins, and whole grains are nutrient-dense foods that contribute several chemical components to support brain structures and physiological functions [7]. Several of these foods contain bioactive compounds that act at the cellular level to modulate gene expression [8].

The outbreak of the global pandemic COVID-19 has produced a sudden disruption in people’s routines with waves of uncertainties and pronounced anxiety. Factors such as sickness, loss of lives and jobs, shift to remote working, and adaptation to a new norm came with a hefty toll on mental health [9,10,11]. However, the catalysts for the decline in mental health may be well related to alterations in dietary and sleep routines and changes in physical exercise patterns. With the modification in sleep schedule comes the loss of meal routines as well. Breakfast was no longer an essential early morning meal for those who were accustomed to eating within a few hours of waking up [12,13]. Breakfast is considered an essential meal of the day, and skipping it appears to trigger mental distress [14,15]. Leading a healthy lifestyle is associated with a healthy diet and healthy dietary practices, such as eating breakfast [16]. Motivation to exercise has been linked to the quality and quantity of food intake [14,17], which was disrupted during lockdowns. Therefore, this change in motivation level may have also contributed to mental status. 

During lockdowns, physical exercise routines were interrupted for many [18]. Stereotypically, people are accustomed to going to the gym to work out; however, unwillingly many elected to exercise at home. Therefore, exercising under stress seems to negate the beneficial effects of physical exercise [19]. The convenience of home exercise led some individuals to increase physical exercise frequency, whereas others lost the motivation to work out. In part, the loss of the weekly structure prevented people from relaxing during weekends. Characteristically, weekdays are for productivity and weekends are times used for revitalization. As COVID-19 dwelled, many individuals adapted to the new norm by finding creative ways to exercise outdoors or at home. As restrictions were eased or lifted and COVID-19 lingered, people re-evaluated their lifestyle standards, and many individuals had to reconsider their physical exercise routine and locations.

Mental distress, such as anxiety and depression, appears to impact men and women differentially with twice the risk in women [20]. Although COVID-19 was associated with increased mental health incidences generally, the literature has done little to describe the sex-related differences in relation to physical exercise, which makes it an unexplored area of research. The differential risk for mental distress may be very likely due to the dimorphic state of the human brain. There are sex-related differences in the density of brain connectivity, regional volumes, and recruitment of neural circuitry under certain tasks [21,22,23]. These morphological variances may explain both these sex-related behavioral and emotional regulation disparities.

Therefore, the purpose of this study was to assess the effect of physical exercise frequency on the mental health of men and women during the three stages of COVID-19: before COVID-19, during COVID-19 (lockdowns), and late COVID-19 (after easing restrictions) while considering the time of the week. Another purpose was to generate mental distress predictive models during similar conditions for future reference. To our knowledge, this is the first study that considers these parameters characterized by differences in biological sexes. Findings from this work may provide the insight needed to optimize mental health during different stages of pandemics by customizing physical exercise frequencies based on gender and time of the week.

## 2. Materials and Methods

### 2.1. Data Set

The study involves a de-identified secondary data collected in the Health and Wellness Studies department at Binghamton University. Data were collected between September 2018 and November 2021 from a baseline mental health status, self-rated survey questionnaire of 2370 individuals who completed the survey. The questionnaire includes 41 variables representative of demographic characteristics, education, dietary behaviors, diet, sleeping and physical exercise pattern, and mental health status.

### 2.2. Data Collection and Analysis

Data collection was through several online platforms targeting college students and older adults. Mental distress was assessed using the Kessler Psychological Distress Scale (K-6), which reflects the degree of non-specific mental distress such as feeling Nervous, Depressed, Worthless, Restless/Fidgety, Hopeless, and Feeling everything was an effort. The score assigned to K-6 responses was None of the time = 0; A little of the time = 1; Some of the time = 2; Most of the time = 3 and All of the time = 4 (Kessler et al., 2003). Therefore, each variable ranged between 0 and 4. The sum of the total scores was used to represent the level of mental distress with variables ranging between 0 and 24. For machine learning modeling, K-6 scores were categorized into 4 levels of mental distress. The scores of 0 were assigned to” No Mental Distress”; 1–5 to” Low Mental Distress”; 6–10 to” Moderate Distress”; and 11 to 24 to” Severe Mental Distress” [24]. Data analysis was conducted in R version 3.6.1., R Foundation for Statistical Computing, Vienna Austria. 

### 2.3. Physical Exercise Assessment

For physical exercise, the data includes frequency of exercising (per week), exercise duration (minutes per day), walking and driving distance to the gym (in minutes), level of athletics (none, recreational or professional), and type of exercise (cardiovascular or strength). There was a question on the “difficulty of exercising before and during COVID-19 quarantine” as well. There was 49 missing records in the dataset accounting for approximately 2.068% of the responses. Therefore, after data cleaning, 2320 records were used for the regression analysis. For this study, exercise frequency includes both cardiovascular and strength exercises. 

### 2.4. Timeframes Assessed

Data were partitioned into three different periods. Before 19 March 2020 is considered before COVID-19, between 19 March 2020 and 1 May 2021 is considered during the COVID-19 lockdowns, based on when most countries enforced a lockdown, and after 1 May 2021 is considered late COVID-19.

### 2.5. Categorization of Exercise Frequency

Less than two times a week were classified as low exercise frequency, between 2 to 4 times a week was considered moderate frequency, and 4 times or more was classified as high frequency.

### 2.6. Ordinal Logistic Regression

To estimate the impact of COVID-19 on an individual’s mental distress, an event-based difference-in-difference study based on the exercise pattern was used in STATA 17.0. Since the dependent variable, mental distress, is a categorical variable with 4 levels, an Ordinal Logistic Regression was performed to analyze factors related to mental distress before, during, and late COVID-19 stratified by gender, weekday/weekend, and a combination of both. The independent variables used in the Ordinal Logistic Regression were breakfast, sleep duration, caffeine, high glycemic food, dairy, meat, seafood, fast food, and exercise type. In this regression model, the focus was to investigate the different frequencies of exercise in relation to mental distress during the three temporal stages of COVID-19. The mathematical formula for ordinal logistic regression, with the interaction term, can be written as Equation (1):

(1) $$\begin{array}{cc} y=\beta_{0} & +\;\beta_{1}X_{\mathrm{COVID19 era}}\times X_{\mathrm{exercise frequency}}+\beta_{2}X_{\mathrm{breakfast frequency}}\\ & +\;\beta_{3}X_{\mathrm{sleep duration}}+\beta_{4}X_{\mathrm{caffeine intake}}\\ & +\;\beta_{5}X_{\mathrm{meat consumption}}+\beta_{6}X_{\mathrm{HGI food consumption}}\\ & +\;\beta_{7}X_{\mathrm{dairu consumption}}+\beta_{8}X_{\mathrm{sea food consumption}}\\ & +\;\beta_{9}X_{\mathrm{fast food consumption}}+\beta_{10}X_{\mathrm{exercise type}}+\varepsilon \end{array}$$

### 2.7. Margins or Predicted Probabilities 

In regression analysis, fitting models are used to predict the outcome of the response variable. In non-linear models with ordinal or categorical response variables, the interest is in making predictions for each outcome. Margins calculate statistics such as the average predicted probability and predictions for each outcome of an ordinal or a categorical dependent variable and let us interpret the models in the probability scale. Adjusted predictions (predictive margins) define values for the independent variables in the model and compute the probability of the event occurring for an individual who has those values. A decision tree describing the adjusted margins of the regression model with severe mental distress outcomes before, during, and late COVID-19 among men and women was used. Mental distress has been utilized as an example to illustrate the work of margins. Since the interest is in mental distress during any of the COVID-19 periods, at the second level, the three branches represent before, during, and late COVID-19, respectively. At the third level, there are other three branches for each of the COVID-19 periods representing physical exercise frequency during each of the COVID-19 eras. The fourth level represents the margins (probability) of being in the severe mental distress category, based on each physical exercise frequency during each of the COVID-19 periods. The purpose of this step was to develop predictive models to describe mental health with different physical exercise frequencies based on gender and time of the week. 

## 3. Results

Table 1 provides descriptive demographic data representing the study cohort. Among respondents, 68.46% were females, about 92% were between the ages of 18 and 29 years, mostly from North America, and the highest majority had completed high school or earned a college degree. 

**Figure 1:**
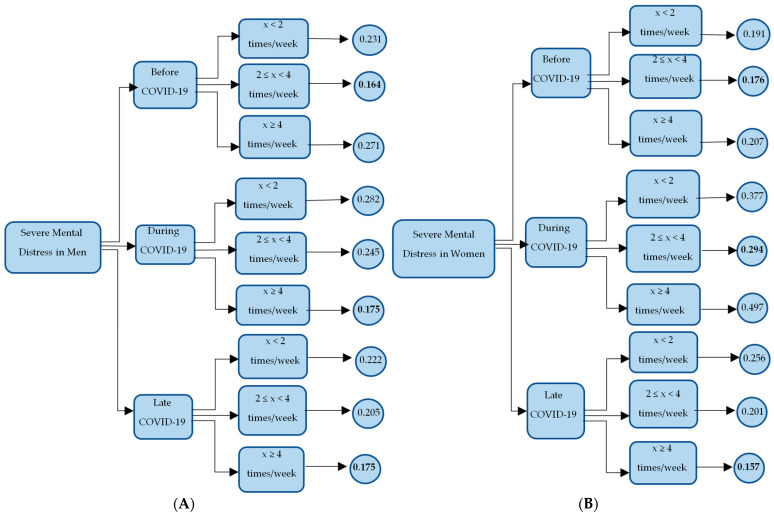
(**A**)—Decision Tree of the adjusted margins (probability) for severe mental distress on men. (**B**)—Decision Tree of the adjusted margins (probability) for severe mental distress on women. Bolded nodes represent the lowest probability and italic nodes represent the highest probability. During COVID-19: during lockdowns; After COVID-19: After COVID-19 ease of restrictions.

The decision tree (Figure 1) visualized the probabilities of being in each category of mental distress for men and women. Based on the decision tree results, men and women who were moderately active before COVID-19 were less likely to experience mental distress; whereas sedentary men and very active women were more likely to experience mental distress. It is interesting to note that COVID-19 changed these equations for men but not for women during the lockdowns. However, by late COVID-19, very active men and women were less likely to experience mental distress.

### 3.1. Regression Analysis Results: Physical Exercise Frequency and Mental Distress among Men and Women

The regression models were used to study the direct effect of physical exercise frequencies on mental distress levels. When no other independent variables were considered, no specific exercise frequency during any of the COVID-19 periods was associated with the K-6 total score among men. This suggests that no specific frequency of exercise during any of the three periods directly contributed to the mental health of men. The results for women were different and interesting. Although women revealed similar findings before COVID-19, there is an obvious trend reflecting the modulatory effect of the pandemic on exercise and mental health. During COVID-19 lockdown, all physical exercise frequencies were significantly associated with mental distress. During late COVID-19 after the ease of restrictions, low exercise levels were associated with mental distress in women (Table 2 and Table 3).

### 3.2. Predictive Probabilities

The next step was to elucidate the findings from each outcome by generating predictive probabilities for moderate and severe mental distress categories after controlling for dietary factors and sleep. All predictive probabilities tables with descriptive numeric data and corresponding figures are in Supplementary Materials. Table 4 and Table 5 represent a summary of the findings.

#### 3.2.1. Probability of Being in the Moderate Mental Distress Category for Men

Men with moderate physical exercise frequency before COVID-19 and those with high physical exercise frequency during lockdowns and late COVID-19 have the lowest probability of being in the moderate mental distress group. On the other hand, the highest probability of being in the moderate mental distress group belongs to men with a low physical exercise frequency during all three COVID-19 periods (Table 4 and Table S1; Figure S1 L).

#### 3.2.2. Probability of Being in the Severe Mental Distress Category for Men

The lowest probability for men to be in the severe mental distress category was associated with moderate exercise frequency before COVID-19, and high physical exercise frequency during the lockdown and during the late COVID-19 period. It is worth noting that this is a parallel trend to the moderate mental distress group. Similarly, to the moderate mental distress category, men with low physical exercise frequency have the highest probability of being in the severe mental distress group, during all three COVID-19 eras (Table 4 and Table S1; Figure S1 R).

#### 3.2.3. Probability of Being in the Moderate Mental Distress Category for Women

A comparable finding was noted for women in the moderate mental distress category. Before COVID-19, women with moderate physical exercise frequency and, those with high physical exercise frequency during and late COVID-19 have the lowest probability of being in the moderate mental distress group. On the other hand, the highest probability of being in the moderate mental distress level belongs to women with high physical exercise frequency and moderate physical exercise frequency during and late COVID-19 (Table 4).

#### 3.2.4. Probability of Being in the Severe Mental Distress Category for Women

The lowest probability of being in the severe mental distress category corresponds to women with moderate physical exercise frequency before and during COVID-19, and with high physical exercise frequency during late COVID-19. Those with moderate physical exercise frequency before and during COVID-19, and with high physical exercise frequency during late COVID-19, have the lowest probability of being in the severe mental distress group. Women with high physical exercise frequency before and during COVID-19 lockdowns, and with low physical exercise frequency in late COVID-19 have the highest probability of being in the severe mental distress group (Table 4 and Table S2, Figure S1 R).

### 3.3. The Impact of Exercise and Time of the Week

#### Regression Results 

No specific physical exercise frequency before COVID-19 is associated with mental distress; however, during COVID-19 lockdowns, low physical exercise frequency and moderate levels are statistically significant with mental distress (*p* < 0.001, and *p* = 0.008, respectively). Low physical exercise frequency during late COVID-19 is statistically significant with mental distress (*p* < 0.001) during weekdays (Table 5). For weekends, low physical exercise frequency is significant during COVID-19. All other physical exercise frequencies are not significant. The results support our hypothesis that time of the week may contribute to mental health, which prompted a gender-based investigation (Table 6).

### 3.4. Predictive Probabilities 

Table 7 represents the probability of being in moderate or severe mental distress categories based on physical exercise frequency during weekdays and weekends, respectively. 

#### 3.4.1. During Weekdays

Before COVID-19, individuals with a moderate physical exercise frequency, and high physical exercise frequency during and late COVID-19 have the lowest probability of being in the moderate mental distress group. A similar trend was detected for the severe mental distress analysis. On the other hand, the highest probability of being in the moderate and severe mental distress category belongs to individuals with a high physical exercise frequency before COVID-19 and low physical exercise levels during the lockdowns and late COVID-19 (Table 7 and Table S3; Figure S2 L).

#### 3.4.2. During Weekends

High physical exercise frequency before COVID-19, moderate to high physical exercise during COVID-19, and high physical exercise levels produced the lowest probability of being in the moderate mental distress group. The lowest probability of being in the severe mental distress category relates to those with a high physical exercise frequency for all three COVID-19 periods. In contrast, individuals with moderate physical exercise frequency before COVID-19, and low physical exercise levels during the lockdowns and late COVID-19 had the highest probability of being in the moderate and severe mental distress categories (Table 8 and Table S4; Figure S2 R).

### 3.5. The Impact of Physical Exercise Frequency and Time of the Week with Respect to Gender

#### 3.5.1. Regression Results

##### Men during Weekends

Before and during the COVID-19 lockdown, the frequency of physical exercise during weekends had a significant impact on mental health. For instance, before COVID-19 only moderate and high physical exercise levels were associated with mental distress; however, during COVID-19 lockdown all physical exercise frequencies may impact mental wellbeing (Table 9).

##### Men during Weekdays

Interestingly, weekdays produced different results. Before COVID-19, moderate physical exercise levels were associated with mental distress and no specific physical exercise frequency had an effect during the COVID-19 lockdown (Table 10 and Table S6; Figure S3 L).

##### Women during Weekends 

Surprisingly the effect of exercising on women’s mental wellbeing was more significant on weekdays than on weekends. During COVID-19 lockdown, only low physical exercise frequency was associated with mental distress on weekends (Table 11).

##### Women during Weekdays 

All physical exercise frequencies were associated with women’s mental distress during COVID-19 lockdown, and only low physical exercise levels produced a significant effect on late COVID-19 during weekdays (Table 12).

Taken together, during lockdown weekdays women experienced higher distress regardless of physical exercise frequency, a mirror image of what men experienced during weekends. On the other hand, weekends were not stressful for women except for those with sedentary lifestyle.

#### 3.5.2. Predictive Probabilities 

Table 13 and Table 14 display the probabilities of being in moderate or severe mental distress while considering physical exercise frequency during weekends and weekdays, respectively, among men and women. 

##### Men during Weekends

The lowest probability of men being in both moderate and severe mental distress groups belongs to those with low physical exercise frequency before COVID-19 and high physical exercise levels during and late COVID-19 (Table 13 and Table S5; Figure S3 R).

##### Men during Weekdays

A common trend was detected for moderate and severe mental distress. Before COVID-19, moderate physical exercise frequency was associated with the lowest probability; however, during and late COVID experienced a shift. Nevertheless, a different pattern was noted for the highest probabilities of moderate versus severe mental distress before COVID-19 (Table 14 and Table S6; Figure S3 L).

##### Women during Weekends

The lowest probability of being in the moderate mental distress group belongs to those with high physical exercise frequency before COVID-19, and moderate exercise frequency levels during the lockdowns and late COVID-19. The lowest probability of a woman being in the severe mental distress group shows a similar trend except for late COVID-19 (Table 13 and Table S7; Figure S4 R). 

##### Women during Weekdays

The lowest probability of being in the moderate mental distress group belongs to those with high physical exercise frequency before COVID-19, and moderate exercise frequency levels during the lockdown and late COVID-19. The lowest probability of being in the severe mental distress group belongs to those with moderate physical exercise frequency before COVID-19, and high levels during the lockdown and late COVID-19 (Table 14 and Table S8; Figure S4 L). 

## 4. Discussion

The purpose of this study was to investigate the effect of COVID-19 on the relationship between physical exercise and mental distress among men and women at two different times of the week. The COVID-19 pandemic was divided into lockdown (during COVID-19) and late COVID-19 after the ease of restrictions (late COVID-19). Before COVID-19 analysis was included as a basis for comparison. Another purpose was to generate prognostic statistical models predictive of mental distress during the different stages of a pandemic to be potentially used prophylactically to support mental health with precision. In addition, a few hypotheses were tested in this study as a proof-concept to support future research in mental health.

The first step was to provide a baseline for the dataset used by determining the highest and lowest probabilities of experiencing severe mental distress during COVID-19 based on physical exercise frequencies among men and women. Few insights were generated from the decision tree. Different physical exercise frequencies are associated with mental distress among men and women before and during the periods of the pandemic, which is a potential proof of concept that customization of physical exercise frequency recommendations is necessary for improving mental wellbeing of men and women. This finding is in line with a previously published report that suggested that customization of dietary and lifestyle factors may be needed to improve mental wellbeing among these cohorts [6]. The lowest probabilities of experiencing mental distress among men and women during the pandemic resulted in differential patterns of physical exercise frequencies. Falkner et al. reported that women engaged less in high-intensity physical exercise but increased their low-intensity workouts during the lockdown [25] potentially to improve their mental wellbeing. On the other hand, it seems that men during quarantine, more likely than women, exercised regularly; and those who led a sedentary lifestyle were more likely to experience mental distress [26]. These results support our findings which reveal that COVID-19 lockdown produced a higher need for men to exercise to experience mental wellbeing, whereas it required women to maintain a moderate physical exercise frequency to support their mental health. Nienhuis and Lesser described that women who were physically active during the lockdown scored higher on emotional and psychological wellbeing scales [27]. However, the authors did not differentiate between moderate and vigorous physical activity. Interestingly, our results suggest that being active during late COVID-19 is associated with the lowest probability of experiencing mental distress among men and women.

Another noteworthy observation is that often low physical exercise frequency increases the probability of mental distress in men, whereas high exercise frequency was often associated with the highest probability of mental distress in women. A cross-sectional study assessing records of 1·2 million individuals in the USA between 2011 and 2015 reported that exercise produced a U-shape effect on mental health. No exercise and too much exercise were detrimental to mental health [28]. However, the study did not categorize exercise frequency based on the mental health of men and women.

The results from the regression analyses along with the predictive probabilities support the notion that women’s mental wellbeing is more sensitive to physical exercise frequencies than men’s during the different periods of the pandemic. The regression models also demonstrated that time of the week is an essential factor to consider when assessing physical exercise frequency and mental health.

### 4.1. The Modulatory Effect of COVID-19 on Physical Exercise Frequency and Mental Distress

The regression analysis controlled for confounding factors such as diet and sleep. Interestingly, no differences were reported before COVID-19, which suggests that physical exercise alone is not the major modulator of mood. The benefit of physical exercise on mental health may be through its mediation effect on food. By changing physiological settings, physical exercise might enhance the beneficial effect of nutrient-dense food and reduce the negative impact of low-quality food [6,29].

For men, no physical exercise frequency was associated with mental distress during all three periods, which proposes that COVID-19 had no effect on the modulatory role of physical exercise on mental distress and that physical exercise has no direct significant impact on men’s mental health. However, women’s regression results were multifaceted. All physical exercise frequencies were associated with mental distress during the lockdown. COVID-19 had a significant effect on the modulatory role of physical exercise on mental distress in women and physical exercise may not possess a calming effect during very stressful times such as the pandemic lockdown. COVID-19 affected women’s mental health more profoundly than that of men [30], and many may have forced themselves to work out. Exercising without motivation, comparable to involuntary exercise in female lab animals [5], may reduce the levels of neurotrophic factors that support a positive mood, which may explain our results.

The next step was to investigate physical exercise frequency and its impact on mental distress concerning the time of the week to test the hypothesis that the loss of the weekly routine during the pandemic may have disrupted the modulatory effect of physical exercise on mental health. Time of the week has a potential impact on mental health due to the stress of the workload that may lead to mental distractions and emotional exhaustion [31]. Weekends are typically used to rest and recharge, which help with continued productivity at work. The shift seen during COVID-19 in physical exercise frequency in relation to the time of the week and mental distress may be in part related to loss of daily routine structure or change in work or sleep schedules that may have affected physical exercise quality and frequency [32].

Results from the regression analysis revealed that during the lockdown and late COVID-19 weekdays and weekends, a sedentary lifestyle was associated with mental distress. This finding supports our hypothesis that loss of daily routine may have impacted physical exercise frequency and increased the risk of mental distress.

However, when the analysis was repeated for men and women, interesting results surfaced. There was a differential pattern of mental distress associated with the time of the week potentially experienced during the lockdown. During lockdown weekends, men were more likely to have been mentally distressed at any physical exercise frequency, whereas women were more likely to experience severe mental distress during lockdown weekdays, at any physical exercise level.

For women, lockdown weekends seem to be less distressful, and presumably, active women were able to relax during downtime. However, during lockdown weekdays, all physical exercise frequencies were associated with a higher risk for mental distress. Weekdays for women mean juggling responsibilities, kids homeschooling, and experiencing a greater workload overall with disrupted routines [32]. Toward the late COVID-19, sedentary women felt the brunt of the pandemic and/or may have experienced weight gain, therefore those who were active in late COVID-19 had a lower risk of mental distress. It is estimated that close to 50% of people gained weight during the lockdown [33], with a higher percentage among women [34]. Weight gain is typically associated with poor body image and low self-esteem. Therefore, the association between high physical exercise frequency and the lowest probability of mental distress in men and women may be linked to improvement in their mental wellbeing due to motivation to lose weight.

The gender difference in the Hypothalamic-Pituitary-Adrenal (HPA) axis activation that modulates the stress response may be at the heart of these differential findings. Gonadal steroid hormones as well as psychoneuroimmunological and neuroanatomical differences between men and women contribute to the differential HPA activity [35]. A dysfunctional HPA axis is typically associated with several mental health ailments [36].

### 4.2. Predictive Models for Future Pandemics

After controlling for diet and sleep, moderately active men who increase the frequency of physical exercise during a pandemic improve their mental wellbeing, and those who lead a sedentary lifestyle may increase their risk of having moderate or severe mental distress. Conversely, active women who remain active are less likely to experience mental distress during a pandemic. Those who are active before the pandemic and reduce their physical exercise frequency will increase their risk of mental distress. When taken all together, being active during a pandemic is more likely to improve mental wellbeing in men and women, which supports a study by Nie et al. [37] that described that moderate physical activity significantly improved mental health in both sexes. Our study adds that women may feel mentally distressed if they go beyond their comfort level.

During lockdown weekends, sedentary men who increase physical exercise frequency or active men who remain active will reduce their chances of experiencing mental distress. During lockdown weekdays, moderately active men who physically exercise frequently will decrease their risk of mental distress. In essence, men need to increase their physical exercise frequency during a pandemic to reduce their risk of mental distress regardless of the time of the week. Conversely, the latter has a great influence on women’s mental wellbeing. During lockdown weekends, active women who decrease their physical exercise frequency or moderately active women who maintain the same level of physical exercise during a pandemic will reduce their chance of experiencing mental distress. During weekdays, there were some conflicting predictions that could be potentially explained by how women perceive physical exercise; therefore, customized recommendations are necessary. If physical exercise is a stressor, reducing frequency may help reduce the risk of mental distress. If physical exercise is a de-stressor, upping exercise frequency may help improve mental wellbeing. However, during the late pandemic, sedentary life may promote a risk of mental distress potentially due to several factors such as social isolation, weight gain, and prolonged low mood; therefore, being active at that point is necessary to improve mental wellbeing. In contrast, active women who reduce their physical exercise frequency will reduce their chance of experiencing mental distress. Nevertheless, overall, women should remain moderately active to reduce their risk of mental distress during a pandemic.

### 4.3. Reason for Physical Exercise

Before COVID-19, the highest risk of mental distress was associated with a sedentary lifestyle. However, COVID-19 changed the modulatory effect of physical exercise on mental health. Although physical activity generally decreased during the pandemic [38], men and women may have exercised for different reasons. One potential explanation for this observation is that men tend to associate physical exercise with a higher quality of life [39], whereas women describe that moderate physical exercise intensity improves the quality of life [40], which explains several of our findings. These conclusions reflect the need for physical exercise regimen adjustment to optimize mental wellbeing during a pandemic. However, the different physical exercise frequencies associated with better mood suggest that stressful times may increase the need for higher physical exercise frequency to modulate mental health among men, whereas a moderate level is enough for women to feel emotionally well. The fact that higher physical exercise frequency may be associated with better mood during the late pandemic period could be due to the return to some exercising normalcy by going back to gyms, which may be associated with increased socializing as well.

## 5. Conclusions and Future Direction

COVID-19 produced an obvious change in the modulatory effect of physical exercise on mental health with a gender-difference pattern. In general, women’s mental health was impacted by COVID-19 and thus needed fine-tuning of physical exercise frequencies to optimize their mental health. In addition, having a sedentary lifestyle overall during COVID-19 may have increased the risk of significantly impacting mental health in both men and women. Moreover, men’s mental health was more disturbed by the pandemic mostly during weekends, whereas women’s mental health was affected by COVID-19 mainly during weekdays. These results provide proof of concept that customization of physical exercise frequency based on gender, time of the week as well as the stage of the pandemic is needed to optimize mental health during periods of calamity. This study, similar to many recent ones, confirms that stratification by gender is needed in scientific research. Larger-scale investigations are needed to confirm these findings and build off these results by incorporating confounding factors such as diet, sleep, and social determinants.

## Figures and Tables

**Table 1:** Descriptive statistics of demographic features (N = 2321).

Feature	Percentage	Number of Observation
**Gender**		
Males	31.54%	732
Females	68.46%	1589
**Age (Years)**		
Under 18	0.95%	22
18–29	91.68%	2128
30–39	2.41%	56
40–49	1.46%	34
above 50	3.50%	81
**Region**		
North America	97.37%	2260
Europe	1.21%	28
MENA	0.26%	6
Asia	0.34%	8
South America	0.22%	5
Australia	0.34%	8
Africa	0.26%	6
**Education**		
Less than high school	1.08%	25
High school	53.34%	1238
College	40.42%	938
Masters	4.09%	95
Doctoral	1.07%	25

**Table 2:** The effect of the COVID-19 era and exercise frequency on mental distress among men.

COVID-19 Era	Exercise Frequency	Coefficient	Standard Error	z	*p* > |z|	[95% Confidence Interval]	
Before COVID-19	x < 2 times per week	Baseline					
	2 ≤ x < 4 times per week	−0.430	0.455	−0.950	0.344	−1.323	0.462
	x ≥ 4 times per week	−0.080	0.517	−0.150	0.878	−1.093	0.934
During COVID-19	x < 2 times per week	0.311	0.373	0.830	0.405	−0.421	1.042
	2 ≤ x < 4 times per week	0.106	0.369	0.290	0.774	−0.617	0.829
	x ≥ 4 times per week	−0.224	0.379	−0.590	0.555	−0.968	0.519
Late COVID-19	x < 2 times per week	−0.026	0.457	−0.060	0.954	−0.923	0.870
	2 ≤ x < 4 times per week	−0.195	0.456	−0.430	0.669	−1.088	0.698
	x ≥ 4 times per week	−0.350	0.446	−0.790	0.432	−1.225	0.524

**Table 3:** The effect of the COVID-19 era and exercise frequency on mental distress among women.

COVID-19 Era	Exercise Frequency	Coefficient	Standard Error	z	*p* > |z|	[95% Confidence Interval]	
Before COVID-19	x < 2 times per week	Baseline					
	2 ≤ x < 4 times per week	−0.104	0.187	−0.560	0.578	−0.471	0.263
	x ≥ 4 times per week	0.121	0.268	0.450	0.653	−0.405	0.646
During COVID-19	**x < 2 times per week**	**1.022**	**0.161**	**6.370**	**0.000**	**0.708**	**1.337**
	**2 ≤ x < 4 times per week**	**0.609**	**0.170**	**3.580**	**0.000**	**0.276**	**0.943**
	**x ≥ 4 times per week**	**1.586**	**0.269**	**5.900**	**0.000**	**1.059**	**2.114**
Late COVID-19	**x < 2 times per week**	**0.430**	**0.220**	**1.960**	**0.050**	**−0.001**	**0.860**
	2 ≤ x < 4 times per week	0.053	0.236	0.230	0.822	−0.409	0.515
	x ≥ 4 times per week	−0.227	0.415	−0.550	0.584	−1.040	0.585

Bolded rows represent significant features.

**Table 4:** Predictive probabilities of moderate and severe mental distress levels by physical exercise frequency and COVID19- era in men and women.

	**Lowest Probability of** **Severe MD**		**Highest Probability of** **Severe MD**	
	*Men*	*Women*	*Men*	*Women*
*Before COVID-19*	Moderate	Moderate	Low	High
*During COVID-19*	High	Moderate	Low	High
*Late COVID-19*	High	High	Low	Low
	**Lowest Probability of** **Moderate MD**		**Highest Probability of** **Moderate MD**	
	*Men*	*Women*	*Men*	*Women*
*Before COVID-19*	Moderate	Moderate	Low	High
*During COVID-19*	High	High	Low	Moderate
*Late COVID-19*	High	High	Low	Low

MD: Mental Distress; During COVID-19: during lockdowns; Late COVID-19: After COVID-19 ease of restrictions.

**Table 5:** The effect of COVID-19 and exercise frequency on mental distress during weekdays.

COVID-19 Era	Exercise Frequency	Coefficient	Standard Error	z	*p* > |z|	[95% Confidence Interval]	
Before COVID-19	x < 2 times per week	Baseline					
	2 ≤ x < 4 times per week	−0.350	0.201	−1.750	0.081	−0.744	0.043
	x ≥ 4 times per week	0.073	0.271	0.270	0.788	−0.457	0.603
During COVID-19	**x < 2 times per week**	**0.787**	**0.167**	**4.720**	**0.000**	**0.460**	**1.114**
	**2 ≤ x < 4 times per week**	**0.449**	**0.169**	**2.650**	**0.008**	**0.117**	**0.781**
	x ≥ 4 times per week	0.334	0.203	1.650	0.099	−0.063	0.732
Late COVID-19	**x < 2 times per week**	**0.428**	**0.211**	**2.030**	**0.043**	**0.014**	**0.842**
	2 ≤ x < 4 times per week	−0.012	0.218	−0.050	0.956	−0.439	0.415
	x ≥ 4 times per week	−0.275	0.272	−1.010	0.312	−0.808	0.258

Bolded rows represent significant features.

**Table 6:** The effect of the COVID-19 era and physical exercise frequency on mental distress during weekends.

COVID-19 Era	Exercise Frequency	Coefficient	Standard Error	z	*p* > |z|	[95% Confidence Interval]	
Before COVID-19	x < 2 times per week	Baseline					
	2 ≤ x < 4 times per week	0.473	0.342	1.380	0.166	−0.197	1.143
	x ≥ 4 times per week	0.329	0.483	0.680	0.495	−0.617	1.275
During COVID-19	**x < 2 times per week**	**1.145**	**0.300**	**3.810**	**0.000**	**0.557**	**1.734**
	2 ≤ x < 4 times per week	0.406	0.354	1.150	0.251	−0.287	1.099
	x ≥ 4 times per week	0.394	0.473	0.830	0.406	−0.534	1.322
Late COVID-19	x < 2 times per week	−0.092	0.573	−0.160	0.873	−1.214	1.031
	2 ≤ x < 4 times per week	−0.456	0.806	−0.570	0.572	−2.035	1.124
	x ≥ 4 times per week	−2.581	2.248	−1.150	0.251	−6.987	1.824

Bolded rows represent significant features.

**Table 7:** Predictive probabilities of moderate and severe mental distress levels by physical exercise frequency and COVID-19 era during weekdays.

	**Lowest Probability**	**Highest Probability**
	*Moderate MD*	*Moderate MD*
*Before COVID-19*	Moderate	High
*During COVID-19*	High	Low
*Late COVID-19*	High	Low
	**Lowest Probability**	**Highest Probability**
	*Severe MD*	*Severe MD*
*Before COVID-19*	Moderate	High
*During COVID-19*	High	Low
*Late COVID-19*	High	Low

MD: Mental distress; During COVID-19: during lockdowns; Late COVID-19: After COVID-19 ease of restrictions.

**Table 8:** Predictive probabilities of moderate and severe mental distress levels by physical exercise frequency and COVID-19 era during weekends.

	**Lowest Probability**	**Highest Probability**
	*Moderate MD*	*Moderate MD*
*Before COVID-19*	High	Moderate
*During COVID-19*	Moderate- High	Low
*Late COVID-19*	High	Low
	**Lowest Probability**	**Highest Probability**
	*Severe MD*	*Severe MD*
*Before COVID-19*	High	Moderate
*During COVID-19*	High	Low
*Late COVID-19*	High	Low

MD: Mental distress; During COVID-19: during lockdowns; Late COVID-19: After COVID-19 ease of restrictions.

**Table 9:** The effect of the COVID-19 era and physical exercise frequency on mental distress in men during weekends.

COVID-19 Era	Exercise Frequency	Coefficient	Standard Error	z	*p* > |z|	[95% Confidence Interval]	
Before COVID-19	x < 2 times per week	Baseline					
	**2 ≤ x < 4 times per week**	**2.522**	**1.132**	**2.230**	**0.026**	**0.304**	**4.741**
	**x ≥ 4 times per week**	**3.305**	**1.295**	**2.550**	**0.011**	**0.766**	**5.844**
During COVID-19	**x < 2 times per week**	**2.806**	**0.898**	**3.120**	**0.002**	**1.045**	**4.566**
	**2 ≤ x < 4 times per week**	**1.875**	**0.979**	**1.920**	**0.055**	**−0.043**	**3.794**
	**x ≥ 4 times per week**	**2.131**	**0.994**	**2.140**	**0.032**	**0.184**	**4.079**
Late COVID-19	x < 2 times per week	1.441	1.449	0.990	0.320	−1.398	4.280
	2 ≤ x < 4 times per week	1.367	1.570	0.870	0.384	−1.710	4.444
	x ≥ 4 times per week	−1.493	3.001	−0.500	0.619	−7.376	4.389

Bolded rows represent significant features.

**Table 10:** The effect of COVID-19 era and physical exercise frequency on mental distress in men during weekdays.

COVID-19 Era	Physical Exercise Frequency	Coefficient	Standard Error	z	*p* > |z|	[95% Confidence Interval]	
Before COVID-19	x < 2 times per week	Baseline					
	**2 ≤ x < 4 times per week**	**−1.179**	**0.534**	**−2.210**	**0.027**	**−2.225**	**−0.133**
	x ≥ 4 times per week	−0.799	0.599	−1.330	0.183	−1.974	0.376
During COVID-19	x < 2 times per week	−0.450	0.439	−1.020	0.306	−1.310	0.411
	2 ≤ x < 4 times per week	−0.358	0.430	−0.830	0.404	−1.201	0.484
	x ≥ 4 times per week	−0.732	0.444	−1.650	0.099	−1.603	0.138
Late COVID-19	x < 2 times per week	−0.619	0.519	−1.190	0.232	−1.636	0.397
	2 ≤ x < 4 times per week	−0.645	0.512	−1.260	0.207	−1.648	0.358
	x ≥ 4 times per week	−0.864	0.498	−1.730	0.083	−1.839	0.112

Bolded rows represent significant features.

**Table 11:** The effect of COVID-19 era and physical exercise frequency on mental distress in women during weekends.

COVID-19 Era	Exercise Frequency	Coefficient	Standard Error	z	*p* > |z|	[95% Confidence Interval]	
Before COVID-19	x < 2 times per week	Baseline					
	2 ≤ x < 4 times per week	0.267	0.376	0.710	0.477	−0.469	1.004
	x ≥ 4 times per week	−0.176	0.546	−0.320	0.747	−1.246	0.895
During COVID-19	**x < 2 times per week**	**0.781**	**0.344**	**2.270**	**0.023**	**0.107**	**1.455**
	2 ≤ x < 4 times per week	0.315	0.420	0.750	0.454	−0.509	1.139
	x ≥ 4 times per week	16.612	804.827	0.020	0.984	−1560.819	1594.043
Late COVID-19	x < 2 times per week	−0.466	0.710	−0.660	0.512	−1.858	0.926
	2 ≤ x < 4 times per week	−0.842	1.186	−0.710	0.478	−3.167	1.483
	x ≥ 4 times per week	0 Empty					

Bolded rows represent significant features.

**Table 12:** The effect of the COVID-19 era and physical exercise frequency on mental distress in women during weekdays.

COVID-19 Era	Exercise Frequency	Coefficient	Standard Error	z	*p* > |z|	[95% Confidence Interval]	
Before COVID	x < 2 times per week	Baseline					
	2 ≤ x < 4 times per week	−0.153	0.222	−0.690	0.490	−0.587	0.281
	x ≥ 4 times per week	0.285	0.317	0.900	0.369	−0.336	0.905
During COVID	**x < 2 times per week**	**1.147**	**0.187**	**6.140**	**0.000**	**0.781**	**1.513**
	**2 ≤ x < 4 times per week**	**0.776**	**0.195**	**3.980**	**0.000**	**0.394**	**1.158**
	**x ≥ 4 times per week**	**1.566**	**0.285**	**5.500**	**0.000**	**1.008**	**2.124**
Late COVID	**x < 2 times per week**	**0.688**	**0.241**	**2.860**	**0.004**	**0.216**	**1.159**
	2 ≤ x < 4 times per week	0.171	0.252	0.680	0.496	−0.322	0.664
	x ≥ 4 times per week	−0.142	0.424	−0.330	0.738	−0.973	0.689

Bolded rows represent significant features.

**Table 13:** Predictive probabilities of moderate and severe mental distress levels by physical exercise frequency and COVID-19 era during weekends among men and women.

	**Lowest Probability of** **Moderate MD**		**Highest Probability of** **Moderate MD**	
	*Men*	*Women*	*Men*	*Women*
*Before COVID-19*	Low	High	High	Moderate
*During COVID-19*	High	Moderate	Low	Low
*Late COVID-19*	High	Moderate	Moderate	High
	**Lowest Probability of** **Severe MD**		**Highest Probability of** **Severe MD**	
	*Men*	*Women*	*Men*	*Women*
*Before COVID-19*	Low	High	High	Moderate
*During COVID-19*	High	Moderate	Low	High
*Late COVID-19*	High	Low	Moderate	High

MD: Mental distress; During COVID-19: during lockdowns; Late COVID-19: After COVID-19 ease of restrictions.

**Table 14:** Predictive probabilities of moderate and severe mental distress levels by physical exercise frequency and COVID-19 era during weekdays among men and women.

	**Lowest Probability of Moderate MD**		**Highest Probability of Moderate MD**	
	*Men*	*Women*	*Men*	*Women*
*Before COVID-19*	Moderate	High	Low	High
*During COVID-19*	High	Moderate	Moderate	High
*Late COVID-19*	High	Moderate	Low	Low
	**Lowest Probability of Severe MD**		**Highest Probability of Severe MD**	
	*Men*	*Women*	*Men*	*Women*
*Before COVID-19*	Moderate	Moderate	Moderate	High
*During COVID-19*	High	High	Moderate	High
*Late COVID-19*	High	High	Low	Low

MD: Mental distress; During COVID-19: during lockdowns; Late COVID-19: After COVID-19 ease of restrictions.

## Data Availability

Data is available upon reasonable request.

## Supplementary Materials

The following supporting information can be downloaded at: https://www.mdpi.com/article/10.3390/nu14132572/s1, Table S1: Predicted margins (probability), *p*-value, and 95% confidence interval of moderate and severe mental distress levels by exercise frequency and COVID-19 era in men. Table S2: Predicted margins (probability), *p*-value, and 95% confidence interval of moderate and severe mental distress levels by exercise frequency and COVID-19 era in Women. Table S3: Predicted margins (probability), *p*-value, and 95% confidence interval of moderate and severe mental distress levels by exercise frequency and COVID-19 era during weekdays. Table S4: Predicted margins (probability), *p*-value, and 95% confidence interval of moderate and severe mental distress levels by exercise frequency and COVID-19 era during weekends. Table S5: Predicted margins (probability), *p*-value, and 95% confidence interval of moderate and severe mental distress levels by exercise frequency and COVID-19 era on men during weekends. Table S6: Predicted margins (probability), *p*-value, and 95% confidence interval of moderate and severe mental distress levels by exercise frequency and COVID-19 era on men during weekdays. Table S7: Predicted margins (probability), *p*-value, and 95% confidence interval of moderate and severe mental distress levels by exercise frequency and COVID-19 era on women during weekdays. Table S8: Predicted margins (probability), *p*-value, and 95% confidence interval of moderate and severe mental distress levels by exercise frequency and COVID-19 era on women during weekends. Figure S1: Left: Predicted margins (probability) of each mental distress level by exercise frequency and COVID-19 duration in men, Right: Predicted margins (probability) of each mental distress level by exercise frequency and COVID-19 duration in women. Figure S2: Left: Predicted margins (probability) of each mental distress level by exercise frequency and COVID-19 duration during weekdays, Right: Predicted margins (probability) of each mental distress level by exercise frequency and COVID-19 duration during weekends. Figure S3: Left: Predicted margins (probability) of each mental distress level by exercise frequency and COVID-19 duration in men during weekdays, Right: Predicted margins (probability) of each mental distress level by exercise frequency and COVID-19 duration in men during weekends. Figure S4: Left: Predicted margins (probability) of each mental distress level by exercise frequency and COVID-19 duration in women during weekdays, Right: Predicted margins (probability) of each mental distress level by exercise frequency and COVID-19 duration in women during weekends. Supplementary Materials contains details the numerical tables of margin analyses with the corresponding figures. All tables include a brief summary of the main findings.
