# Supplementary material for: The Impact of the Different Stages of COVID-19, Time of the Week and Exercise Frequency on Mental Distress in Men and Women
Source: Nutrients. 2022 Jun 21;14(13):2572. doi: 10.3390/nu14132572 (PMC9268137; doi:10.3390/nu14132572)
Supplement: Supplementary file 1 [file nutrients-14-02572-s001.zip › nutrients-1763574-supplementary.pdf]

	COVID-19 Era	Exercise Frequency	Margin	Standard Error	z	P>|z|	[95% confidence	Interval]
Moderate Mental Distress	Before COVID-19	*x < 2 times a week*	*0.247*	*0.027*	*9.230*	*0.000*	*0.195*	*0.299*
		**2 ≤ x < 4 times a week**	**0.212**	**0.029**	**7.190**	**0.000**	**0.154**	**0.270**
		x ≥ 4 times a week	0.241	0.030	7.960	0.000	0.182	0.301
	During COVID-19	*x < 2 times a week*	*0.262*	*0.019*	*14.180*	*0.000*	*0.226*	*0.299*
		2 ≤ x < 4 times a week	0.252	0.018	14.070	0.000	0.217	0.287
		**x ≥ 4 times a week**	**0.219**	**0.019**	**11.310**	**0.000**	**0.181**	**0.257**
	Late COVID-19	*x < 2 times a week*	*0.244*	*0.026*	*9.260*	*0.000*	*0.192*	*0.295*
		2 ≤ x < 4 times a week	0.236	0.027	8.880	0.000	0.184	0.288
		**x ≥ 4 times a week**	**0.219**	**0.027**	**8.100**	**0.000**	**0.166**	**0.272**
Severe Mental Distress	Before COVID-19	*x < 2 times a week*	*0.231*	*0.058*	*3.980*	*0.000*	*0.117*	*0.344*
		**2 ≤ x < 4 times a week**	**0.164**	**0.041**	**4.010**	**0.000**	**0.084**	**0.244**
		x ≥ 4 times a week	0.217	0.062	3.520	0.000	0.096	0.337
	During COVID-19	*x < 2 times a week*	*0.282*	*0.034*	*8.370*	*0.000*	*0.216*	*0.348*
		2 ≤ x < 4 times a week	0.245	0.025	9.700	0.000	0.196	0.295
		**x ≥ 4 times a week**	**0.175**	**0.024**	**7.220**	**0.000**	**0.127**	**0.222**
	Late COVID-19	*x < 2 times a week*	*0.222*	*0.053*	*4.210*	*0.000*	*0.119*	*0.326*
		2 ≤ x < 4 times a week	0.205	0.047	4.330	0.000	0.112	0.298
		**x ≥ 4 times a week**	**0.175**	**0.040**	**4.410**	**0.000**	**0.097**	**0.252**

Table S1: Predicted margins (probability), P-value, and 95% confidence interval of moderate and severe mental distress levels by exercise frequency and COVID-19 era in men. **Moderate exercise frequency before COVID-19, and high exercise frequency during and late COVID-19 are associated with the lowest probability to be in the moderate and severe mental distress levels. Low exercise frequency before, during, and late COVID-19 are associated with the highest probability to be in the moderate and severe mental distress levels.**

	COVID-19 Era	Exercise Frequency	Margin	Standard Error	Z	P>|z|	[95% confidence Interval]	
Moderate Mental Health Disorder	Before COVID-19	x < 2 times a week	0.323	0.016	20.720	0.000	0.293	0.354
		**2 ≤ x < 4 times a week**	**0.313**	**0.015**	**20.270**	**0.000**	**0.283**	**0.344**
		x ≥ 4 times a week	*0.332*	*0.020*	*16.580*	*0.000*	*0.293*	*0.371*
	During COVID-19	x < 2 times a week	0.353	0.013	28.070	0.000	0.329	0.378
		2 ≤ x < 4 times a week	*0.356*	*0.013*	*28.250*	*0.000*	*0.331*	*0.381*
		**x ≥ 4 times a week**	**0.321**	**0.023**	**14.160**	**0.000**	**0.276**	**0.365**
	Late COVID-19	*x < 2 times a week*	*0.349*	*0.015*	*23.400*	*0.000*	*0.320*	*0.379*
		2 ≤ x < 4 times a week	0.329	0.019	17.490	0.000	0.292	0.366
		**x ≥ 4 times a week**	**0.300**	**0.041**	**7.300**	**0.000**	**0.219**	**0.380**
Severe Mental Health Disorder	Before COVID-19	x < 2 times a week	0.191	0.020	9.660	0.000	0.153	0.230
		**2 ≤ x < 4 times a week**	**0.176**	**0.019**	**9.140**	**0.000**	**0.138**	**0.213**
		x ≥ 4 times a week	*0.207*	*0.036*	*5.720*	*0.000*	*0.136*	*0.278*
	During COVID-19	x < 2 times a week	0.377	0.021	17.900	0.000	0.335	0.418
		**2 ≤ x < 4 times a week**	**0.294**	**0.021**	**14.080**	**0.000**	**0.253**	**0.334**
		x ≥ 4 times a week	*0.497*	*0.052*	*9.520*	*0.000*	*0.395*	*0.600*
	Late COVID-19	*x < 2 times a week*	*0.256*	*0.032*	*7.920*	*0.000*	*0.193*	*0.320*
		2 ≤ x < 4 times a week	0.201	0.030	6.720	0.000	0.143	0.260
		**x ≥ 4 times a week**	**0.157**	**0.049**	**3.180**	**0.001**	**0.060**	**0.254**

Table S2: Predicted margins (probability), P-value, and 95% confidence interval of moderate and severe mental distress levels by exercise frequency and COVID-19 era in Women. **Moderate exercise frequency before COVID-19, and high exercise frequency during and late COVID-19 are associated with the lowest probability to be in the moderate mental distress level, and moderate exercise frequency before, and during COVID-19, and high exercise frequency late COVI D-19 are associated with the lowest probability to be in the severe mental distress level. High exercise frequency before COVID-19, and moderate exercise frequency during and late COVID-19 are associated with the highest probability to be in the moderate mental distress level, and High exercise frequency before and during COVID-19, and low exercise frequency in late COVID-19 era are associated with the highest probability to be in the severe mental distress levels.**

	COVID-19 Era	Exercise Frequency	Margin	Standard Error	z	P>|z|	[95% confidence	Interval]
Moderate Mental Health Disorder	Before COVID-19	x < 2 times a week	0.304	0.015	20.090	0.000	0.274	0.334
		**2 ≤ x < 4 times a week**	**0.271**	**0.017**	**16.070**	**0.000**	**0.238**	**0.304**
		x ≥ 4 times a week	*0.308*	*0.020*	*15.740*	*0.000*	*0.269*	*0.346*
	During COVID-19	*x < 2 times a week*	*0.334*	*0.011*	*29.380*	*0.000*	*0.311*	*0.356*
		2 ≤ x < 4 times a week	0.329	0.011	28.740	0.000	0.307	0.352
		**x ≥ 4 times a week**	**0.322**	**0.013**	**24.690**	**0.000**	**0.297**	**0.348**
	Late COVID-19	*x < 2 times a week*	*0.328*	*0.013*	*25.510*	*0.000*	*0.303*	*0.353*
		2 ≤ x < 4 times a week	0.304	0.016	18.550	0.000	0.272	0.336
		**x ≥ 4 times a week**	**0.276**	**0.025**	**11.240**	**0.000**	**0.228**	**0.324**
Severe Mental Health Disorder	Before COVID-19	x < 2 times a week	0.205	0.023	8.940	0.000	0.160	0.250
		**2 ≤ x < 4 times a week**	**0.155**	**0.019**	**8.200**	**0.000**	**0.118**	**0.192**
		x ≥ 4 times a week	*0.213*	*0.037*	*5.760*	*0.000*	*0.140*	*0.285*
	During COVID-19	*x < 2 times a week*	*0.348*	*0.021*	*16.960*	*0.000*	*0.308*	*0.389*
		2 ≤ x < 4 times a week	0.281	0.018	15.640	0.000	0.246	0.317
		**x ≥ 4 times a week**	**0.251**	**0.026**	**9.620**	**0.000**	**0.200**	**0.302**
	Late COVID-19	*x < 2 times a week*	*0.273*	*0.031*	*8.770*	*0.000*	*0.212*	*0.335*
		2 ≤ x < 4 times a week	0.205	0.026	7.790	0.000	0.154	0.257
		**x ≥ 4 times a week**	**0.162**	**0.030**	**5.330**	**0.000**	**0.102**	**0.222**

Table S3: Predicted margins (probability), P-value, and 95% confidence interval of moderate and severe mental distress levels by exercise frequency and COVID-19 era during weekdays. **Moderate exercise frequency before COVID-19, and high exercise frequency during and late COVID-19 are associated with the lowest probability to be in the moderate and severe mental distress levels. High exercise frequency before COVID-19, and low exercise frequency during and late COVID-19 are associated with the highest probability to be in the moderate and severe mental distress levels.**

	COVID-19 Era	Exercise Frequency	Margin	Standard Error	z	P>|z|	[95% confidence	Interval]
Moderate Mental Health Disorder	Before COVID-19	x < 2 times a week	0.267	0.027	10.060	0.000	0.215	0.319
		2 ≤ x < 4 times a week	*0.287*	*0.023*	*12.230*	*0.000*	*0.241*	*0.333*
		**x ≥ 4 times a week**	**0.262**	**0.035**	**7.430**	**0.000**	**0.193**	**0.331**
	During COVID-19	*x < 2 times a week*	*0.297*	*0.023*	*12.880*	*0.000*	*0.252*	*0.342*
		**2 ≤ x < 4 times a week**	**0.278**	**0.024**	**11.610**	**0.000**	**0.231**	**0.325**
		**x ≥ 4 times a week**	**0.278**	**0.031**	**9.010**	**0.000**	**0.217**	**0.338**
	Late COVID-19	*x < 2 times a week*	*0.246*	*0.048*	*5.090*	*0.000*	*0.151*	*0.340*
		2 ≤ x < 4 times a week	0.210	0.074	2.840	0.005	0.065	0.355
		**x ≥ 4 times a week**	**0.049**	**0.094**	**0.530**	**0.599**	**-0.135**	**0.233**
Severe Mental Health Disorder	Before COVID-19	x < 2 times a week	0.200	0.035	5.680	0.000	0.131	0.269
		2 ≤ x < 4 times a week	*0.251*	*0.039*	*6.360*	*0.000*	*0.173*	*0.328*
		**x ≥ 4 times a week**	**0.190**	**0.057**	**3.310**	**0.001**	**0.077**	**0.302**
	During COVID-19	*x < 2 times a week*	*0.413*	*0.040*	*10.370*	*0.000*	*0.335*	*0.492*
		2 ≤ x < 4 times a week	0.225	0.036	6.180	0.000	0.153	0.296
		**x ≥ 4 times a week**	**0.224**	**0.061**	**3.680**	**0.000**	**0.105**	**0.343**
	Late COVID-19	*x < 2 times a week*	*0.164*	*0.064*	*2.550*	*0.011*	*0.038*	*0.289*
		2 ≤ x < 4 times a week	0.120	0.075	1.600	0.111	-0.027	0.267
		**x ≥ 4 times a week**	**0.017**	**0.037**	**0.460**	**0.645**	**-0.056**	**0.090**

Table S4: Predicted margins (probability), P-value, and 95% confidence interval of moderate and severe mental distress levels by exercise frequency and COVID-19 era during weekends. **High exercise frequency before, during and late COVID-19 are associated with the lowest probability to be in the moderate and severe mental distress levels. Moderate exercise frequency before COVID-19, and low exercise frequency during and late COVID-19 are associated with the highest probability to be in the moderate and severe mental distress levels.**

	COVID-19 Era	Exercise Frequency	Margin	Standard Error	z	P>|z|	[95% confidence Interval]	
Moderate Mental Health Disorder	Before COVID-19	**x < 2 times a week**	**0.138**	**0.066**	**2.080**	**0.038**	**0.008**	**0.267**
		2 ≤ x < 4 times a week	0.277	0.053	5.210	0.000	0.172	0.381
		x ≥ 4 times a week	*0.284*	*0.056*	*5.080*	*0.000*	*0.174*	*0.394*
	During COVID-19	*x < 2 times a week*	*0.291*	*0.047*	*6.130*	*0.000*	*0.198*	*0.383*
		2 ≤ x < 4 times a week	0.257	0.049	5.280	0.000	0.162	0.352
		**x ≥ 4 times a week**	**0.247**	**0.051**	**4.860**	**0.000**	**0.147**	**0.347**
	Late COVID-19	x < 2 times a week	0.178	0.113	1.570	0.117	-0.045	0.400
		2 ≤ x < 4 times a week	*0.245*	*0.114*	*2.160*	*0.031*	*0.023*	*0.468*
		**x ≥ 4 times a week**	**0.041**	**0.072**	**0.560**	**0.572**	**-0.101**	**0.182**
Severe Mental Health Disorder	Before COVID-19	**x < 2 times a week**	**0.077**	**0.043**	**1.790**	**0.073**	**-0.007**	**0.161**
		2 ≤ x < 4 times a week	0.246	0.103	2.380	0.017	0.044	0.449
		x ≥ 4 times a week	*0.432*	*0.171*	*2.530*	*0.011*	*0.097*	*0.766*
	During COVID-19	*x < 2 times a week*	*0.371*	*0.073*	*5.070*	*0.000*	*0.227*	*0.514*
		2 ≤ x < 4 times a week	0.199	0.061	3.260	0.001	0.079	0.319
		**x ≥ 4 times a week**	**0.182**	**0.061**	**2.970**	**0.003**	**0.062**	**0.302**
	Late COVID-19	x < 2 times a week	0.106	0.084	1.250	0.210	-0.060	0.271
		2 ≤ x < 4 times a week	*0.179*	*0.170*	*1.050*	*0.293*	*-0.155*	*0.513*
		**x ≥ 4 times a week**	**0.018**	**0.044**	**0.400**	**0.686**	**-0.069**	**0.105**

Table S5: Predicted margins (probability), P-value, and 95% confidence interval of moderate and severe mental distress levels by exercise frequency and COVID-19 era on men during weekends. **Low exercise frequency before COVID-19, and high exercise frequency during and late COVID-19 are associated with the lowest probability to be in the moderate and severe mental distress levels. High exercise frequency before COVID-19, low exercise frequency during COVID-19, and moderate exercise frequency late COVID-19 are associated with the highest probability to be in the moderate and severe mental distress levels.**

	COVID-19 Era	Exercise Frequency	Margin	Standard Error	z	P>|z|	[95% confidence Interval]	
Moderate Mental Health Disorder	Before COVID-19	*$x < 2$ times a week*	*0.268*	*0.021*	*12.950*	*0.000*	*0.228*	*0.309*
		**$2 \leq x < 4$ times a week**	**0.192**	**0.035**	**5.480**	**0.000**	**0.124**	**0.261**
		$x \geq 4$ times a week	0.225	0.037	6.030	0.000	0.152	0.299
	During COVID-19	$x < 2$ times a week	0.249	0.021	11.760	0.000	0.208	0.291
		$2 \leq x < 4$ times a week	*0.255*	*0.019*	*13.160*	*0.000*	*0.217*	*0.293*
		**$x \geq 4$ times a week**	**0.223**	**0.021**	**10.490**	**0.000**	**0.181**	**0.265**
	Late COVID-19	*$x < 2$ times a week*	*0.240*	*0.029*	*8.390*	*0.000*	*0.184*	*0.296*
		$2 \leq x < 4$ times a week	0.239	0.027	8.720	0.000	0.185	0.293
		**$x \geq 4$ times a week**	**0.219**	**0.028**	**7.890**	**0.000**	**0.164**	**0.273**
Severe Mental Health Disorder	Before COVID-19	*$x < 2$ times a week*	*0.324*	*0.083*	*3.910*	*0.000*	*0.161*	*0.486*
		**$2 \leq x < 4$ times a week**	**0.137**	**0.042**	**3.290**	**0.001**	**0.055**	**0.219**
		$x \geq 4$ times a week	0.185	0.062	2.980	0.003	0.063	0.307
	During COVID-19	$x < 2$ times a week	0.238	0.035	6.820	0.000	0.170	0.306
		$2 \leq x < 4$ times a week	*0.254*	*0.027*	*9.300*	*0.000*	*0.201*	*0.308*
		**$x \geq 4$ times a week**	**0.181**	**0.028**	**6.550**	**0.000**	**0.127**	**0.236**
	Late COVID-19	*$x < 2$ times a week*	*0.214*	*0.054*	*3.930*	*0.000*	*0.107*	*0.320*
		$2 \leq x < 4$ times a week	0.212	0.051	4.190	0.000	0.113	0.311
		**$x \geq 4$ times a week**	**0.174**	**0.040**	**4.350**	**0.000**	**0.096**	**0.253**

Table S6: Predicted margins (probability), P-value, and 95% confidence interval of moderate and severe mental distress levels by exercise frequency and COVID-19 era on men during weekdays. **Moderate exercise frequency before COVID-19, and high exercise frequency during and late COVID-19 are associated with the lowest probability to be in the moderate and severe mental distress levels. Low exercise frequency before and late COVID-19, and moderate exercise frequency during COVID-19 are associated with the highest probability to be in the moderate and severe mental distress levels.**

	Covid-19 Era	Exercise Frequency	Margin	Standard Error	z	P>|z|	[95% confidence Interval]	
Moderate Mental Health Disorder	Before Covid	x < 2 times a week	0.328	0.019	17.270	0.000	0.291	0.366
		**2 ≤ x < 4 times a week**	**0.312**	**0.020**	**15.920**	**0.000**	**0.274**	**0.351**
		x ≥ 4 times a week	*0.351*	*0.023*	*15.530*	*0.000*	*0.307*	*0.396*
	During Covid	x < 2 times a week	0.366	0.014	25.530	0.000	0.338	0.394
		2 ≤ x < 4 times a week	*0.372*	*0.014*	*26.010*	*0.000*	*0.344*	*0.400*
		**x ≥ 4 times a week**	**0.341**	**0.023**	**14.670**	**0.000**	**0.295**	**0.386**
	Late Covid	*x < 2 times a week*	*0.370*	*0.015*	*24.470*	*0.000*	*0.340*	*0.399*
		2 ≤ x < 4 times a week	0.345	0.020	17.250	0.000	0.306	0.385
		**x ≥ 4 times a week**	**0.311**	**0.044**	**7.160**	**0.000**	**0.226**	**0.397**
Severe Mental Health Disorder	Before Covid	x < 2 times a week	0.174	0.022	7.910	0.000	0.131	0.217
		**2 ≤ x < 4 times a week**	**0.153**	**0.021**	**7.330**	**0.000**	**0.112**	**0.194**
		x ≥ 4 times a week	*0.214*	*0.044*	*4.850*	*0.000*	*0.128*	*0.301*
	During Covid	x < 2 times a week	0.379	0.024	15.720	0.000	0.332	0.426
		**2 ≤ x < 4 times a week**	**0.303**	**0.024**	**12.830**	**0.000**	**0.257**	**0.350**
		x ≥ 4 times a week	*0.471*	*0.054*	*8.790*	*0.000*	*0.366*	*0.576*
	Late Covid	*x < 2 times a week*	*0.282*	*0.037*	*7.720*	*0.000*	*0.210*	*0.353*
		2 ≤ x < 4 times a week	0.202	0.031	6.520	0.000	0.141	0.262
		**x ≥ 4 times a week**	**0.152**	**0.049**	**3.120**	**0.002**	**0.056**	**0.248**

Table S7: Predicted margins (probability), P-value, and 95% confidence interval of moderate and severe mental distress levels by exercise frequency and Covid-19 era on women during weekdays. **Moderate exercise frequency before COVID-19, and high exercise frequency during and late COVID-19 are associated with the lowest probability to be in the moderate mental distress level, and moderate exercise frequency before and during COVID-19, and high exercise frequency late COVID-19 are associated with the lowest probability to be in the severe mental distress level. High exercise frequency before COVID-19, moderate exercise frequency during COVID-19, and low exercise frequency late COVID-19 are associated with the highest probability to be in the moderate mental distress level, and high exercise frequency before and during COVID-19, and low exercise frequency late COVID-19 are associated with the highest probability to be in the severe mental distress level.**

	Covid-19 Era	Exercise Frequency	Margin	Standard Error	z	P>|z|	[95% confidence	Interval]
Moderate Mental Health Disorder	Before Covid	x < 2 times a week	0.282	0.031	9.230	0.000	0.222	0.342
		2 ≤ x < 4 times a week	*0.297*	*0.027*	*11.070*	*0.000*	*0.244*	*0.349*
		**x ≥ 4 times a week**	**0.270**	**0.039**	**6.970**	**0.000**	**0.194**	**0.346**
	During Covid	*x < 2 times a week*	*0.311*	*0.027*	*11.550*	*0.000*	*0.258*	*0.364*
		**2 ≤ x < 4 times a week**	**0.299**	**0.027**	**11.020**	**0.000**	**0.246**	**0.352**
		x ≥ 4 times a week	0.000	0.000	0.000	0.999	-0.001	0.001
	Late Covid	x < 2 times a week	0.247	0.059	4.200	0.000	0.132	0.362
		**2 ≤ x < 4 times a week**	**0.214**	**0.104**	**2.060**	**0.040**	**0.010**	**0.418**
		x ≥ 4 times a week	*0.282*	*0.031*	*9.230*	*0.000*	*0.222*	*0.342*
Severe Mental Health Disorder	Before Covid	x < 2 times a week	0.213	0.038	5.560	0.000	0.138	0.288
		2 ≤ x < 4 times a week	*0.252*	*0.041*	*6.170*	*0.000*	*0.172*	*0.332*
		**x ≥ 4 times a week**	**0.189**	**0.061**	**3.130**	**0.002**	**0.071**	**0.308**
	During Covid	x < 2 times a week	0.338	0.044	7.710	0.000	0.252	0.424
		**2 ≤ x < 4 times a week**	**0.259**	**0.047**	**5.540**	**0.000**	**0.168**	**0.351**
		x ≥ 4 times a week	*1.000*	*0.000*	*2427.920*	*0.000*	*0.999*	*1.001*
	Late Covid	**x < 2 times a week**	**0.155**	**0.073**	**2.130**	**0.033**	**0.012**	**0.298**
		2 ≤ x < 4 times a week	0.118	0.103	1.150	0.251	-0.083	0.319
		x ≥ 4 times a week	*0.213*	*0.038*	*5.560*	*0.000*	*0.138*	*0.288*

Table S8: Predicted margins (probability), P-value, and 95% confidence interval of moderate and severe mental distress levels by exercise frequency and Covid-19 era on women during weekends. **high exercise frequency before COVID-19, and moderate exercise frequency during and late COVID-19 are associated with the lowest probability to be in the moderate mental distress level, and high exercise frequency before COVID-19, moderate exercise frequency during COVID-19, and low exercise frequency late COVID-19 are associated with the lowest probability to be in the severe mental distress level. Moderate exercise frequency before COVID-19, low exercise frequency during COVID-19, and high exercise frequency late COVID-19 are associated with the highest probability to be in the moderate mental distress level, and moderate exercise frequency before COVID-19, and high exercise frequency during and late COVID-19 are associated with the highest probability to be in the severe mental distress level.**

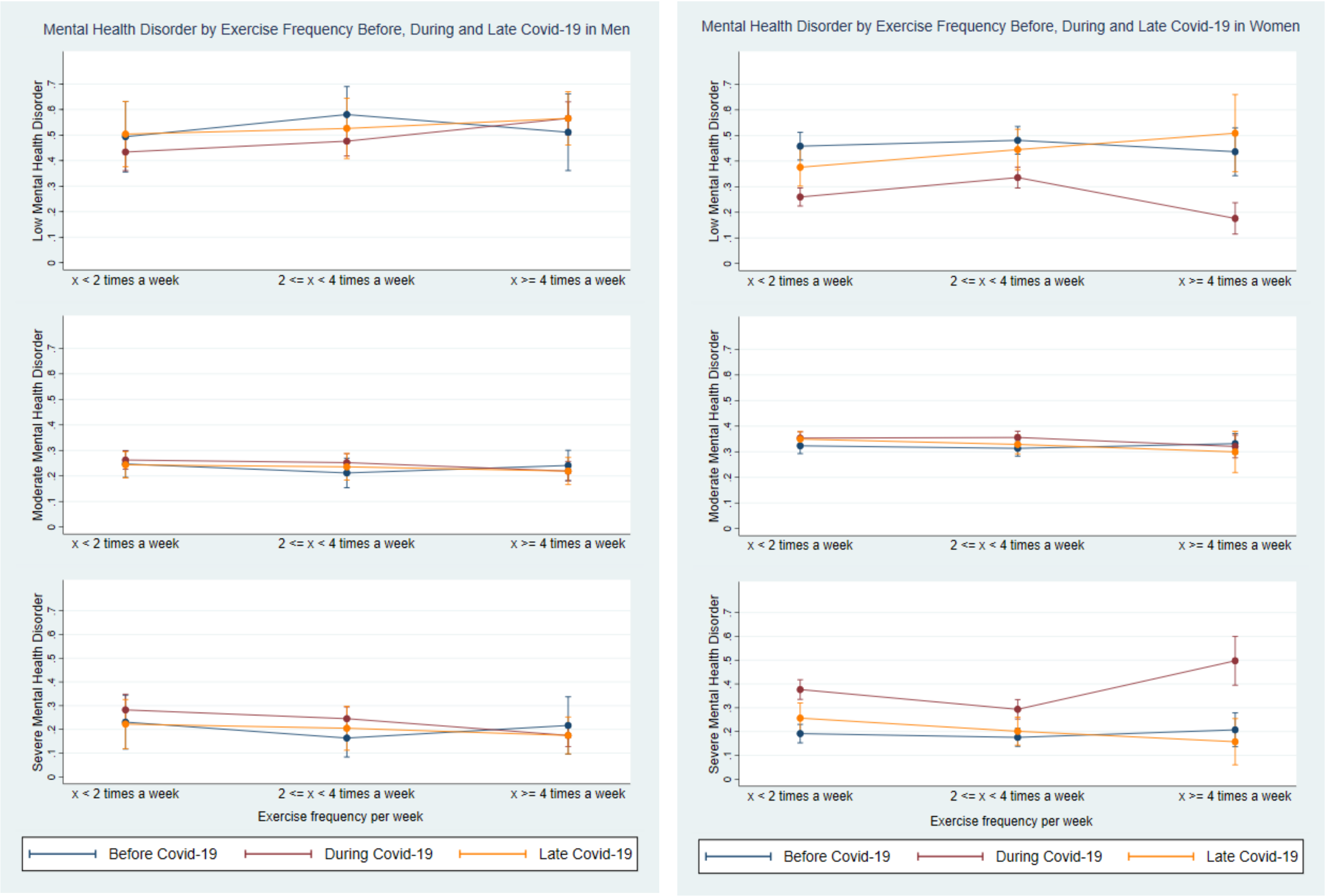

Figure S1: Left: Predicted margins (probability) of each mental distress level by exercise frequency and COVID-19 duration in men - Right: Predicted margins (probability) of each mental distress level by exercise frequency and COVID-19 duration in women

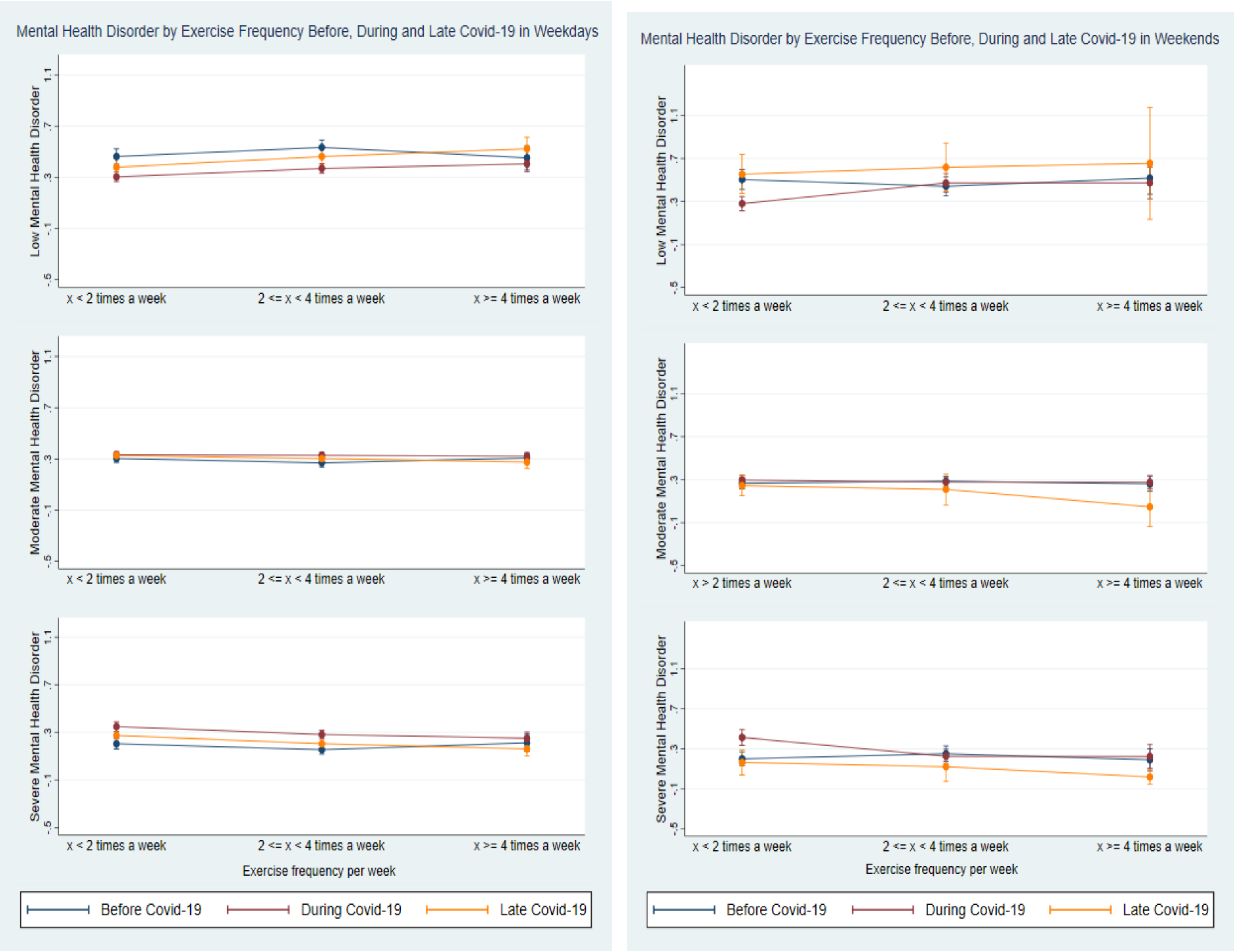

Figure S2: Left: Predicted margins (probability) of each mental distress level by exercise frequency and COVID-19 duration during weekdays - Right: Predicted margins (probability) of each mental distress level by exercise frequency and COVID-19 duration during weekends

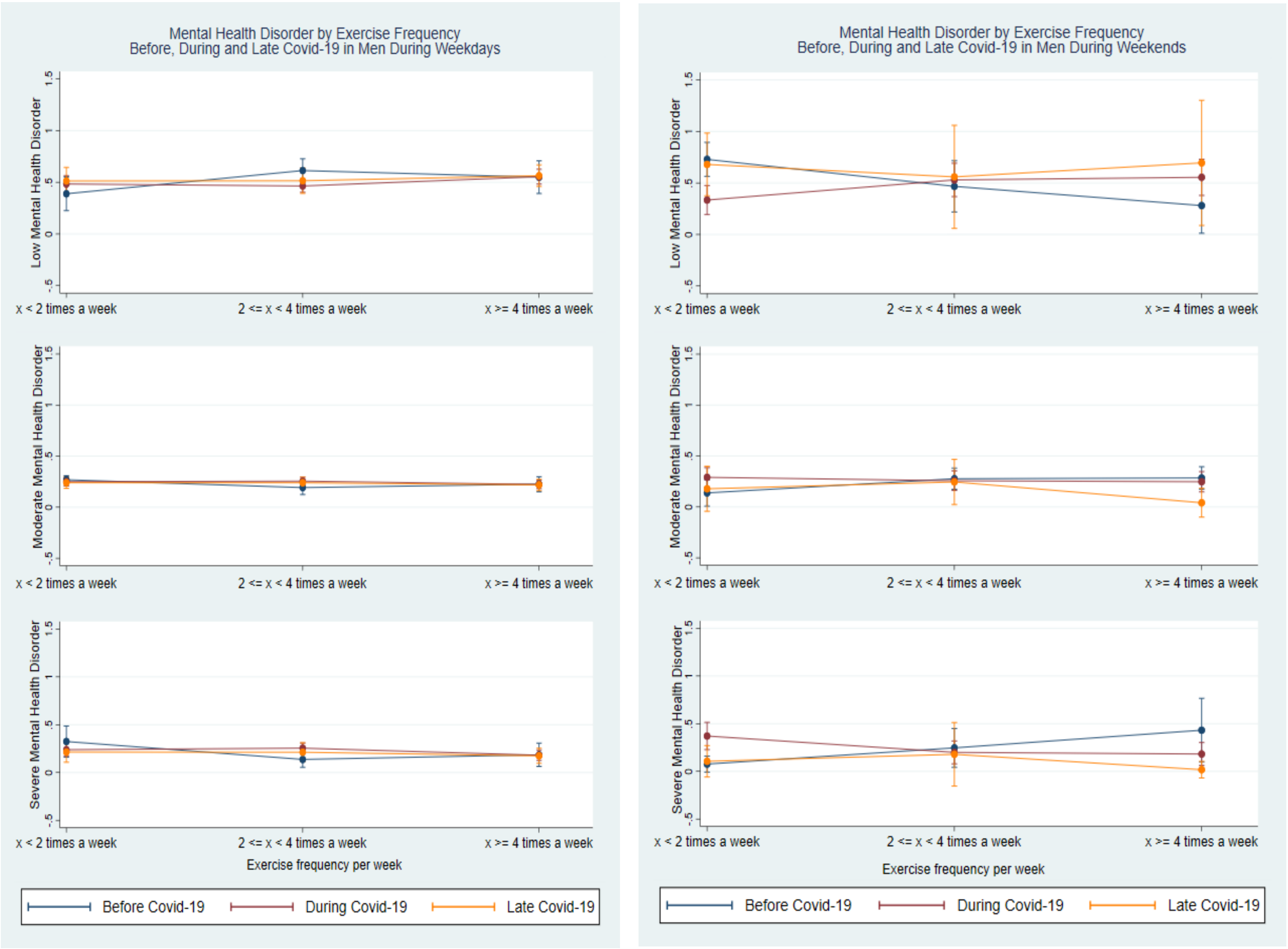

Figure S3: Left: Predicted margins (probability) of each mental distress level by exercise frequency and COVID-19 duration in men during weekdays - Right: Predicted margins (probability) of each mental distress level by exercise frequency and COVID-19 duration in men during weekends

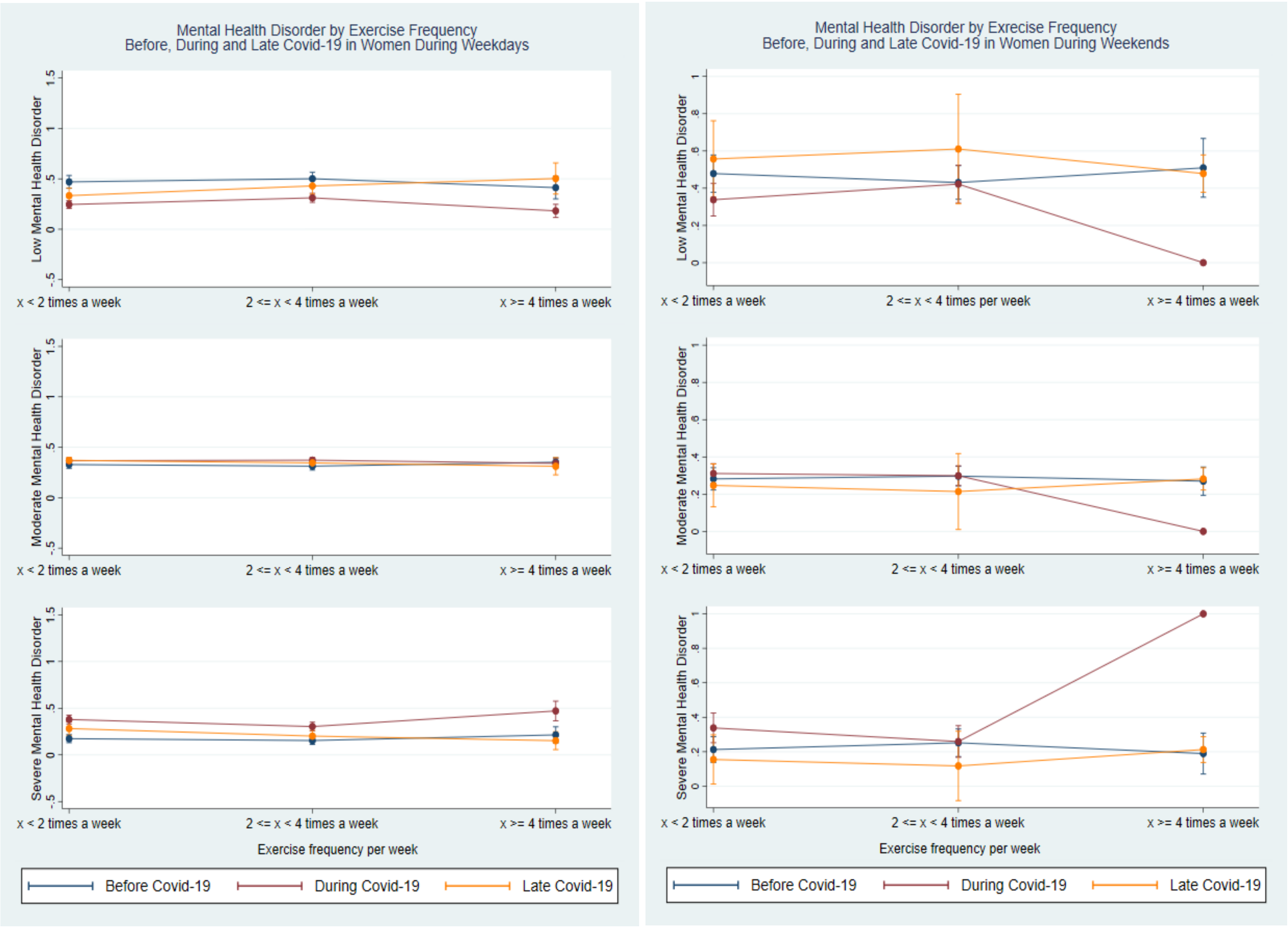

Figure S4: Left: Predicted margins (probability) of each mental distress level by exercise frequency and COVID-19 duration in women during weekdays - Right: Predicted margins (probability) of each mental distress level by exercise frequency and COVID-19 duration in women during weekends
